# ﻿*Planothidiumpseudolinkei* sp. nov. (Bacillariophyta), a new marine monoraphid diatom species from the coast of Guangxi, China

**DOI:** 10.3897/phytokeys.246.128068

**Published:** 2024-09-05

**Authors:** Lang Li, Yu-Yang Huang, Qun-Zhuan Nong, Jun-Xiang Lai, Yu-Hang Li

**Affiliations:** 1 Guangxi Key Laboratory of Marine Environmental Science, Guangxi Academy of Marine Sciences, Guangxi Academy of Sciences, Nanning 530007, China Guangxi Academy of Marine Sciences, Guangxi Academy of Sciences Nanning China; 2 Beibu Gulf Marine Industry Research Institute, Fangchenggang 538000, China Beibu Gulf Marine Industry Research Institute Fangchenggang China; 3 School of Resources, Environment and Materials, Guangxi University, Nanning 530004, China Guangxi University Nanning China; 4 Laboratory of Marine Organism Taxonomy and Phylogeny, Qingdao Key Laboratory of Marine Biodiversity and Conservation, Institute of Oceanology, Chinese Academy of Sciences, Qingdao 266071, China Institute of Oceanology, Chinese Academy of Sciences Qingdao China

**Keywords:** China, Guangxi, monoraphid diatom, new species, *Planothidium*

## Abstract

A new marine monoraphid diatom species, *Planothidiumpseudolinkei***sp. nov.**, is described from the coast of Guangxi, China. The detailed morphology of this epipsammic diatom is studied by using both light and scanning electron microscopy. *P.pseudolinkei* differs from congeners by a combination of morphological features including capitate apices, multiseriate striae, a small central area on the raphe valve and an oblong sinus on the rapheless valve. Ecological preferences of *Planothidium* are also briefly discussed.

## ﻿Introduction

Monoraphid diatoms represent a large assemblage of species which is characterized by the presence of a single raphe system on one valve. Over the past 40 years, 12 marine monoraphid genera have been described: *Bennettella* R.W.Holmes, *Epipellis* R.W.Holmes, *Astartiella* A.Witkowski et al., *Pogoneis* Round & Basson, *Pauliella* Round & Basson, *Vikingea* A.Witkowski et al., *Scalariella* Riaux-Gob., *Madinithidium* A.Witkowski et al., *Majewskaea* Van de Vijver, *Navithidium* Al-Handal & Romero, *Xenobennettella* A.Witkowski & Riaux-Gob. and *Vallithidium* J.A.Nienow & A.K.S.K.Prasad (Holmes 1985; Round and Basson 1997; Witkowski et al. 2000, 2022; Riaux-Gobin et al. 2012, 2013; Desrosiers et al. 2014; Van de Vijver et al. 2020; Al-Handal et al. 2021; Nienow and Prasad 2023). According to Round et al. (1990), all monoraphid species should be included in the order Achnanthales Silva. But Kulikovskiy et al. (2016) suggested that this order was not a monophyletic group.

As a monoraphid diatom genus, *Planothidium* Round & Bukht. was erected and separated from *Achnanthes* Bory based on its morphological features of bi- to multiseriate striae, internally raised virgae, elongated terminal fissures and unilaterally asymmetrical central areas on the rapheless valves in many species (Round and Bukhtiyarova 1996). At present, AlgaeBase lists a total of 123 taxonomically accepted *Planothidium* species and infraspecific names (Guiry and Guiry 2024). Morales (2006) divided this genus into four groups by the features of the central area of the rapheless valve. The first group is commonly represented by *Planothidiumdelicatulum* (Kützing) Round & Bukht., which presents continuous striae on the rapheless valve. The species in second group are characterized by a clear space without any depression at the central area such as in *Planothidiumminutissimum* (Krasske) Morales. The other two groups are marked by a sinus (a rimmed depression) or a cavum (a hood) and represented species are *Planothidiumlanceolatum* (Brébisson ex Kützing) Lange-Bertalot and *Planothidiumfrequentissimum* (Lange-Bertalot) Lange-Bertalot, respectively. However, a molecular investigation of *Planothidium* confirmed two distinct clades within the genus, one of them possessing a sinus or a cavum on the rapheless valve and the other lacking either additional structure (Jahn et al. 2017). So far, Marine and brackish *Planothidium* have been rarely investigated. The current divisions, which are prepared for freshwater species, may not reflect the morphological diversity and phylogenetic relationship of marine and brackish species. Apart from *Planothidium*, the cavum can also be seen in another four monoraphid genera: *Xenobennettella*, *Gliwiczia* Kulikovskiy et al., *Skabitschewskia* Kuliskovskiy & Lange-Bertalot and *Planoplatessa* Kulikovskiy et al. (Kulikovskiy et al. 2022; Witkowski et al. 2022). It was suggested that the cavum might work as a lens for utilization of sunlight in the *Planothidium* cells (Bukhtiyarova and Lyakh 2014). But the function of the sinus is poorly known. Up to now, there are six species of *Planothidium* recorded from marine environments in China (Li 2019; Chen et al. 2022). Most of them live in sandy sediments from coastal areas of Fujian (Chen et al. 2022), no *Planothidium* taxa has been reported from Guangxi.

During a survey of the epipsammic diatom flora along the coast of Guangxi, China, an unknown *Planothidium* species was found on the sand grains from two sampling sites. Under the light microscope (LM), this taxon is very similar to *Planothidiumlinkei* (Hustedt) Lange-Bertalot. But the ultrastructural observations reveal that it is quite different from the latter. Therefore, we describe the new species as *Planothidiumpseudolinkei* sp. nov. brief information on its ecology is also provided.

## ﻿Materials and methods

**Figure 1.**
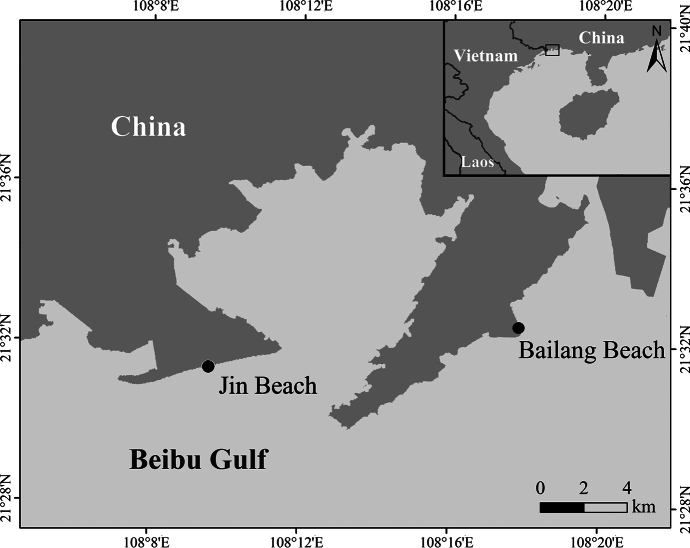
Locations of the sampling sites in this study.

Sampling was conducted along the coast of Fangchenggang City, Guangxi Province, China on 10 January 2021. We selected two locations as sampling sites in our study (Fig. 1). The first location is the Jin Beach (21°31.85′N, 108°10.95′E), which is located at the southern part of Wanwei Island and faces south. The second location is the Bailang Beach (21°32.27′N, 108°17.43′E), which lies in the middle part of Jiangshan Peninsula and faces east. Fangchenggang is situated in the north of Beibu Gulf with an average precipitation of 2362.6 mm (Du et al. 2022). The city has a subtropical monsoon climate and an average temperature ranging from 14 °C to 29 °C (Du et al. 2022). Its coastal region is dominated by irregular diurnal tides with maximum and average tidal ranges exceeding 4.5 m and 2.5 m, respectively (Huang et al. 2023).

At each site, sand samples were scraped by a hand shovel from the low intertidal zone of the beach during low tide and then placed into polyethylene ziplock bags with some air for transporting. Upon return to the laboratory, sub-samples were pretreated in an ultrasonic bath at 700 W for 3 minutes to separate diatoms attached to the sand grains. Prior to observation and analysis, diatom samples were digested with concentrated nitric acid (65%–68%) at 100 °C for 20 minutes to eliminate organic matter. After several rinses with Milli-Q water, cleaned materials were dried onto coverslips and permanently mounted onto slides with Naphrax^®^. Diatoms were examined and identified at a magnification of 1,000× using a Zeiss Imager Z2 (Carl Zeiss, Germany) microscope equipped with differential interference contrast (DIC) and Zeiss Axiocam 512 color digital camera. Measurements were taken from 75 individuals for the new species. Relative abundance data was based on LM counts of at least 1500 valves. Valve ultrastructure was observed and imaged by a Hitachi S-3400 scanning electron microscope (SEM) (Hitachi, Japan) operated at 10 kV and 6 mm working distance. For SEM, small aliquots of suspensions were air-dried onto coverslips or membrane filters attached to aluminum stubs with conductive tape, and then sputter coated with 10 nm of gold in a Hitachi MC 1000.

Terminology follows Morales (2006), Jahn et al. (2017), Van de Vijver et al. (2018) and Van de Vijver and Bosak (2019).

## ﻿Results

### ﻿Division Bacillariophyta

**Class Bacillariophyceae**

**Order Achnanthales**

**Family Achnanthidiaceae**

**Genus *Planothidium***

#### ﻿ Planothidium pseudolinkei

Taxon classificationPlantaeMastogloialesAchnanthaceae

Lang Li, Yuhang Li & Junxiang Lai sp. nov.

5819BBDC-F6BA-5C33-B28D-25D04681CE37

Figs 2 , 3 , 4

##### Holotype.

**Figure 2.**
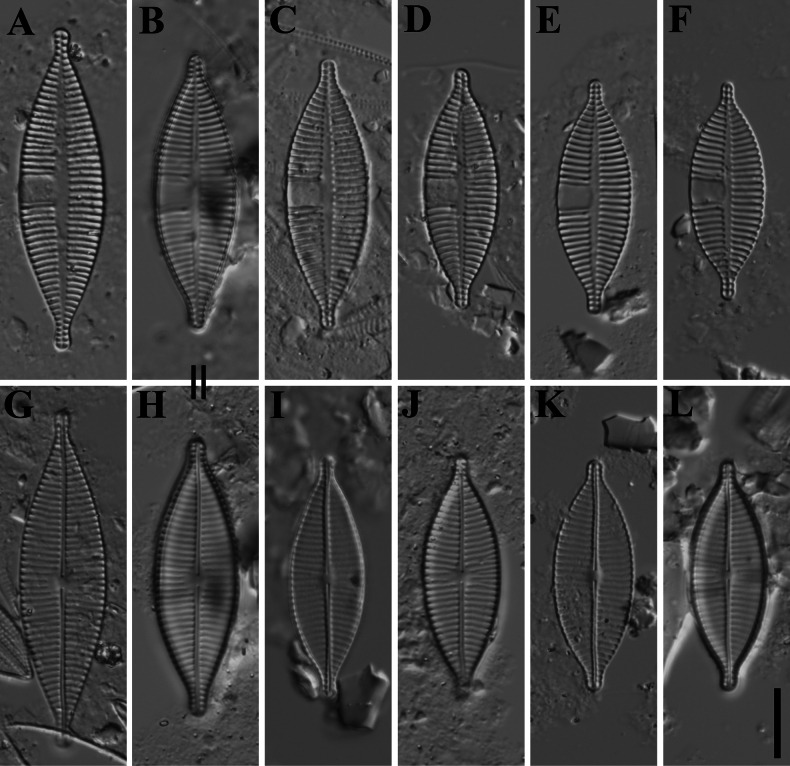
*Planothidiumpseudolinkei* sp. nov. LM **A–F** rapheless valves **G–L** raphe valves **B, H** holotype designated. “=” indicates the different valves of the same frustule. Scale bars: 10 μm.

Slide MBMCAS286907 deposited in the Marine Biological Museum, Chinese Academy of Sciences (MBMCAS), Qingdao, China, represented here by Fig. 2B (Rapheless valve) and Fig. 2H (Raphe valve).

##### Type locality.

Jin Beach, Wanwei Island, Guangxi Province, China, 21°31.85′N, 108°10.95′E, surface sand samples, collected by Lang Li on 10 January 2021.

##### Description.

***LM*** (Fig. 2A–L). Valves lanceolate to elliptic-lanceolate with convex margins and protracted, capitate to rostrate apices. Valve dimensions (n = 75): length 26.5–50.0 μm, width 10.0–13.0 μm. Rapheless valve (Fig. 2A–F): Axial area linear and narrowly lanceolate, slightly expanded in the middle of the valve. Central area asymmetrical with a unilateral large hyaline area; on the opposite side, striae barely shortened near the axial area. Striae weakly radiate at the center and more strongly radiate towards the apices, 11–12 in 10 μm. Raphe valve (Fig. 2G–L): Axial area very narrow, linear, slightly widened near the middle of the valve. Central area very small, slightly transapically expanded, bordered by 3–4 shortened striae on each side. Raphe straight, filiform with slightly enlarged central raphe endings. Terminal fissures not discernible in LM. Striae radiate throughout the entire valve, 12 in 10 μm. Areolae not discernible in LM.

**Figure 3.**
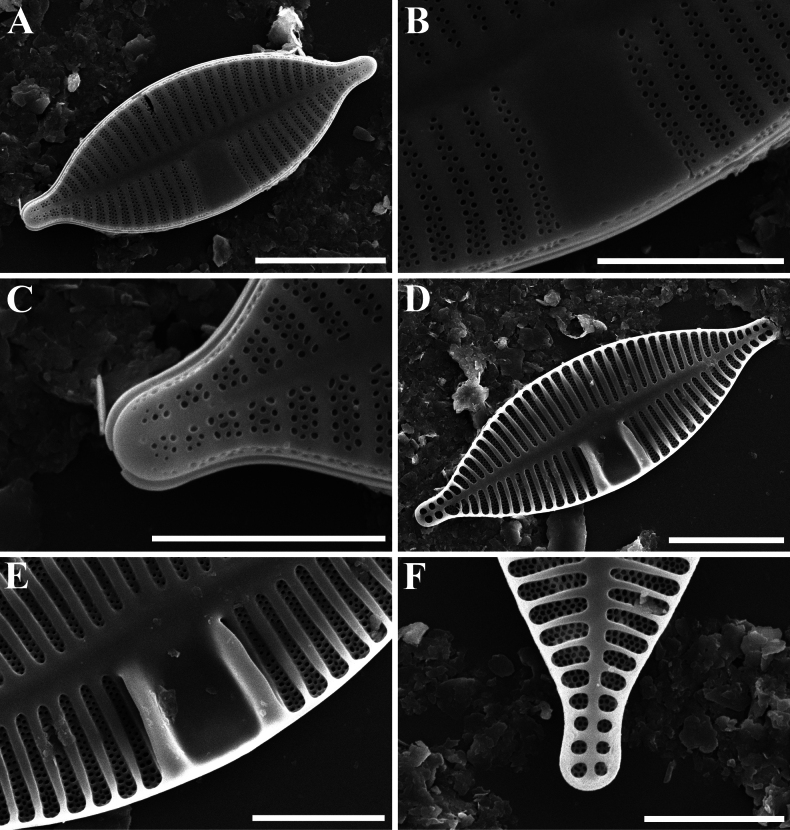
*Planothidiumpseudolinkei* sp. nov. SEM**A** external view of an entire rapheless valve **B** external detail of the interruption of the striae on the rapheless valve **C** external detail of the valve apex **D** internal view of an entire rapheless valve **E** internal detail of the sinus depression **F** internal detail of the valve apex. Scale bars: 10 μm (**A, D**); 5 μm (**B, C, E, F**).

**Figure 4.**
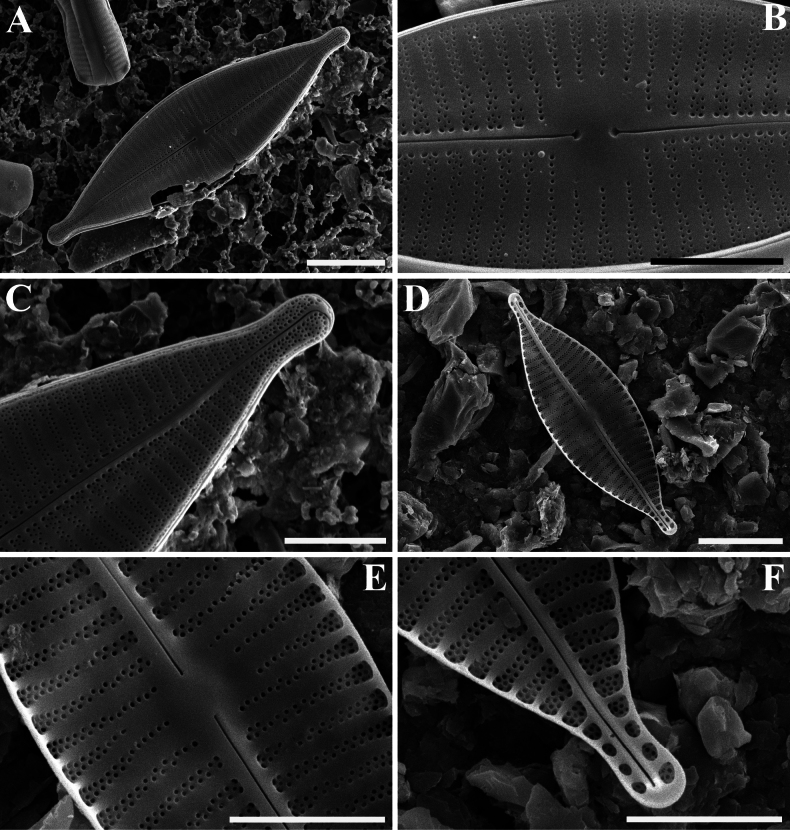
*Planothidiumpseudolinkei* sp. nov. SEM**A** external view of an entire raphe valve **B** external detail of the central area of the raphe valve **C** external detail of the valve apex **D** internal view of an entire raphe valve **E** internal detail of the central area of the raphe valve **F** internal detail of the valve apex. Scale bars: 10 μm (**A, D**); 5 μm (**B, C, E, F**).

***SEM*** (Figs 3A–F, 4A–F). Rapheless valve (Fig. 3A–F): Externally, valve face generally flat, with a slightly linear depression in the axial area (Fig. 3A). Central area large, unilateral (Fig. 3A, B). Striae multiseriate, composed of three rows of small, rounded areolae at the apices, varying from three rows near the valve face/mantle junction to biseriate towards the axial area (Fig. 3B, C). Striae portion near the axial area often composed of one or two areolae (Fig. 3B). Striae extending onto the valve mantle, but interrupted by the valve face-mantle junction (Fig. 3B, C). Internally, axial area elevated and striae sunken between the raised virgae (Fig. 3D–F). A well-developed sinus distinctly present on one side of the central area, with the borders fusing with lateral virgae, forming a deep oblong depression (Fig. 3D, E). Raphe valve (Fig. 4A–F): Externally, valve face flat (Fig. 4A). Striae composed of one to four rows of small, rounded areolae, clearly broader than virgae at the apices (Fig. 4B, C). Striae portion along the axial area composed of two larger areolae than other ones (Fig. 4B, C). Central raphe endings straight, simple, expanded and pore-like (Fig. 4B). Terminal fissures bent, continuing shortly onto the valve mantle (Fig. 4C). Internally, virgae poorly developed (Fig. 4D–F). Raphe hook-shaped, branches situated on a slightly elevated raphe-sternum (Fig. 4D). Terminal raphe endings terminated by small helictoglossae (Fig. 4F).

##### Etymology.

The specific epithet, *pseudolinkei*, was referring to the morphological and habitat similarities with *P.linkei*.

##### PhycoBank registration.

http://phycobank.org/104835.

##### Distribution and ecology.

In addition to the type locality, *P.pseudolinkei* also occurs in the surface sand sample from the Bailang Beach of Jiangshan Peninsula. This taxon is a marine epipsammic diatom. In the type material, *P.pseudolinkei* was very rare and only found in abundance of 0.14%. The associated diatom flora is composed of *Amphora* spp., *Navicula* spp., *Diploneis* spp., *Fallacia* spp., *Cocconeiopsis* spp., *Gyrosigma* sp., *Planothidium* sp., *Anorthoneishummii* Hustedt, *Biremisambigua* (Cleve) D.G.Mann, *Cymatoneismargarita* A.Witkowski, *Moreneiscoreana* J.Park et al. and *Halamphoracoffeiformis* (C.Agardh) Levkov.

## ﻿Discussion

*Planothidium* is a species-rich genus which is widespread in freshwater, brackish and marine environments. Most representatives can be found in freshwater habitats, only a few taxa are marine or brackish species (Marquardt et al. 2021). In marine environments, however, the diversity of *Planothidium* species was suggested to be higher than that which has been found (Riaux-Gobin et al. 2018; Van de Vijver and Bosak 2019; Lai et al. 2021). It has been supported by several recently discovered marine *Planothidium* species, such as *P.galaicum* Álvarez-Blanco & S. Blanco, *P.juandenovense* Riaux-Gob. & A.Witkowski and *P.kaetherobertianum* Van de Vijver & Bosak (Álvarez-Blanco and Blanco 2013; Riaux-Gobin et al. 2018; Van de Vijver and Bosak 2019). Van de Vijver and Bosak (2019) pointed out that most marine and brackish species of the genus *Planothidium* had completely symmetrical central areas. In this genus, the presence/absence of a cavum or sinus is the most important taxonomic criterion, followed by the valve outline and morphometric data (Jahn et al. 2017). But Stancheva et al. (2020) reported that the striae density is often useless for distinguishing similar cavum- or sinus-bearing species. Given the importance of ultrastructural details, the SEM observation is necessary to illustrate the morphology of the *Planothidium* species (Stancheva et al. 2020).

*P.pseudolinkei* has a unique combination of morphological features of capitate apices, multiseriate striae, thickened virgae and internal depressions in the central areas of rapheless valves. All these characters justify its separation from all other species in the genus *Planothidium*. Our new taxon shares some similarities with a few previously described species, such as *P.apiculatum* (R.M.Patrick) Lange-Bertalot, *P.dispar* (Cleve) A.Witkowski et al., *P.iberense* L.Rovira & A.Witkowski, *P.lanceolatoides* (Sovereign) Lange-Bertalot, *P.oculatum* (Hustedt) A.Witkowski et al. and *P.rostrolanceolatum* Van de Vijver et al. A detailed comparison of these taxa is summarized in Table 1. *P.apiculatum* and *P.lanceolatoides* differ from *P.pseudolinkei* by having apiculate apices, a large central area on the raphe valve and a cavum on the rapheless valve (Patrick 1945; Sovereign 1958; Potapova 2010, 2015). In *P.dispar*, the central area on the raphe valve is also moderately large and the striae on the rapheless valve are continuous (Witkowski et al. 2000). *P.iberense* has a smaller cell size (17.0–26.0 μm long, 6.5–9.5 μm wide) and denser striae on both valves (raphe valve: 12–15/10 μm, rapheless valve: 14–16/10 μm), no additional structures are formed in the central area of the rapheless valve (Rovira et al. 2011). *P.oculatum* differs by its smaller cell size (10.0–20.0 μm long, 4.0–5.0 μm wide), denser striae on both valves (raphe valve: 20–24/10 μm, rapheless valve: 12–16/10 μm), broad axial area on the rapheless valve and absence of a sinus (Witkowski et al. 2000). Though *P.rostrolanceolatum* shows a sinus with circular depression on the rapheless valve, it has a large central area on the raphe valve and denser striae on both valves (raphe valve: 13–15/10 μm, rapheless valve: 14–16/10 μm) (Van de Vijver et al. 2013).

**Table 1.** Comparison of morphological characteristics of *Planothidiumpseudolinkei* sp. nov. and similar species.

	*P.apiculatum*	*P.dispar*	*P.iberense*	*P.lanceolatoides*	*P.oculatum*	*P.rostrolanceolatum*	*P.linkei*	*P.pseudolinkei*
Length (μm)	28.0	16.0–55.0	17.0–26.0	22.0–35.0	10.0–20.0	15.0–28.0	34.0–38.0	26.5–50.0
Width (μm)	10.0–11.0	6.0–15.0	6.5–9.5	10.0–12.0	4.0–5.0	5.6–7.8	12.0–15.0	10.0–13.0
Striae in 10 μm (rapheless valve)	10–12	14–20	14–16	11–13	12–16	14–16	11	11–12
Striae in 10 μm (raphe valve)	10	14–20	12–15	11–14	20–24	13–15	11	12
Apices	Apiculate	Rostrate	Broadly rounded, slightly capitate	Apiculate	Slightly rostrate	Rostrate, protracted	Markedly protracted	Protracted, capitate to rostrate
Central area (raphe valve)	Large	Moderately large	Rather large	Large	Small	Large	Large	Small
Axial area (rapheless valve)	Narrow	Narrow	Narrow	Narrow	Broad	Narrow	Narrow	Narrow
Additional structure	Cavum	Absent	Absent	Cavum	Absent	Sinus	Absent	Sinus
Striae pattern	Multiseriate	Unknown	Multiseriate	Multiseriate	Unknown	Multiseriate	Biseriate	Multiseriate
References	Patrick 1945; Potapova 2010	Witkowski et al. 2000	Rovira et al. 2011	Sovereign 1958; Potapova 2015	Witkowski et al. 2000	Van de Vijver et al. 2013	Andrews 1981	This study

*P.pseudolinkei* is easily confused with *P.linkei* under LM, as they have the same valve outlines, overlapping valve dimensions, similar striae densities and unilateral central areas of the rapheless valves. Additionally, both species occur in marine habitats (Andrews 1981). *P.linkei* was originally reported by Schulz (1926) and described as *Achnantheslinkei* Hustedt in Hustedt (1939). Lange-Bertalot transferred it into the genus *Planothidium* (Lange-Bertalot 1999). Recently, Van de Vijver et al. (2018) re-examined the type material of *P.delicatulum* and found that most specimens are *P.linkei*. Based on the LM observations in Hustedt (1939) and Van de Vijver et al. (2018), *P.linkei* has a larger central area on the raphe valve and a narrower unilateral hyaline area on the rapheless valve than those of *P.pseudolinkei*. The ultrastructural features of *P.linkei* was firstly revealed in Andrews (1981) by using SEM. It can be seen that the clear space on the rapheless valve of *P.linkei* is actually a gap between the central striae without any depression (Andrews 1981). Thus *P.linkei* should be belonging to the *P.minutissimum*-group (Morales 2006; Rovira et al. 2011). In addition, the two species can also be distinguished by the striae pattern (multiseriate striae in *P.pseudolinkei* vs. biseriate striae in *P.linkei*). Witkowski et al. (2000) published three *P.linkei* LM photographs (plate 48, figs 42–44). However, the morphology of their specimen does not entirely correspond with the type material, because both raphe and rapheless valves have a relatively large central area (Hustedt 1939; Simonsen 1987; Witkowski et al. 2000). Most likely, these valves belong to an unknown *Planothidium* species, which needs to be further investigated.

*Planothidium* is a typical benthic diatom genus which has diverse ecological preferences, whether in freshwater, brackish or marine habitats (Kulaš et al. 2020). Species within the genus can attach to various inorganic and biotic substrates by their raphe valves (Spaulding et al. 2008; Wetzel et al. 2019; Morais et al. 2020). For example, *P.delicatulum* and *P.deperditum* (Giffen) A.Witkowski et al. are two epipsammic diatom species, *P.galaicum* and *P.hinzianum* C.E.Wetzel et al. are two epiphytic diatom species, and *P.africanum* Van de Vijver et al. and *P.wetzelii* Schimani et al. are two epilithic diatom species (Witkowski et al. 2000; Álvarez-Blanco and Blanco 2013; Wetzel et al. 2019; Juchem et al. 2023; Van de Vijver et al. 2023). Interestingly, Van de Vijver and Bosak (2019) also described an epizoic species, *P.kaetherobertianum*, which was on the carapace of one sea turtle. In the present study, the newly documented species is an epipsammic diatom collected from the intertidal zone in the city of Fangchenggang. As a unique benthic community, epipsammic diatoms are able to adapt to sandy environments with unstable substrates and insufficient nutrients and have only recently started to receive domestic research attention in China, such as Zhao et al. (2017), Zhang et al. (2020), Li et al. (2021) and Liu et al. (2022). Their results had illustrated the biodiversity of diatoms in this specific habitat and improved our understanding of marine epipsammic diatoms in China. We speculate that more newly described epipsammic taxa will be found along with the further investigation of sand samples from different coastal areas in China.

## Supplementary Material

XML Treatment for Planothidium pseudolinkei

## ﻿Additional information

### Author ORCIDs

Lang Li https://orcid.org/0000-0001-5649-1097

Yu-Yang Huang https://orcid.org/0009-0009-7327-1518

Qun-Zhuan Nong https://orcid.org/0009-0004-6646-1528

Jun-Xiang Lai https://orcid.org/0000-0002-7139-1454

Yu-Hang Li https://orcid.org/0000-0002-5546-1774

### Data availability

All of the data that support the findings of this study are available in the main text.
